# Anticonvulsant Activity of Schiff Bases of 3-Amino-6,8-dibromo-2-phenyl-quinazolin-4(3*H*)-ones

**DOI:** 10.4103/0250-474X.70488

**Published:** 2010

**Authors:** P. Paneersalvam, T. Raj, M. P. S. Ishar, B. Singh, V. Sharma, B. A. Rather

**Affiliations:** 1 Department of Pharmaceutical Chemistry, C. L. Baid Mehta College of Pharmacy, Jyothi Nagar, Rajiv Gandhi Salai, Thorapakkam, Chennai - 600 097, India; Bio-Organic and Photochemistry Laboratory, Department of Pharmaceutical Sciences, Guru Nanak Dev University, Amritsar-143 005, India

**Keywords:** Anticonvulsant, maximal electroshock method, Schiff base, Quinazolin-4-(3*H*)-one, 3-aminoquinazolines

## Abstract

Schiff bases (9a-*l*) of 3-amino-6,8-dibromo-2-phenyl-quinazolin-4-(3*H*)-ones (8) with various substituted aldehydes were obtained by refluxing 1:1 molar equivalents of the reactants in dry ethanol for 6 h. The aminoquinazoline (8) was inturn obtained from 3,5-dibromoantharlinic acid via intermediate (7). All the synthesized compounds (9a-l) were evaluated for their anticonvulsant activity on albino mice by maximal electroshock method using phenytoin as a standard. The compound (9l) bearing a cinnamyl function displays a very high activity (82.74 %) at dose level of 100 mg/kg b.w.

Quinazolinones and their Schiff bases are an important class of heterocyclic systems, enjoying considerable interest on account of their diverse range of biological activities[1–9] such as antimicrobial, analgesic and antiinflammatory, anticonvulsant, anticancer, antitubercular, antimalarial, antiviral, antihelmintic and, in particular, very high anticonvulsant activity. Anticonvulsant quinazolinones include methaqualone (1)[10], which acts on voltage dependant sodium channels in a manner similar to rilozole (2)[11,12], 2-(chloromethyl)-1-(4-methoxyphenyl)-quinazolin-4(1*H*)-one (3) and 2-methyl-3-(5-phenyl-4,5-dihydroisoxazoli-3-ylamino)quinazolin-4(3*H*)-one (4, fig. 1). Structural modification of the quinozolines nucleus i.e., introduction of Ph- and -CH_3_group at C2 position[13], bromination of benzene ring at C6 and C8, introduction of various substituted phenyl moieties[14], bridged phenyl rings[15], heterocyclic rings[16] and aliphatic moieties[17] at position-3 are reported to enhance the anticonvulsant activity. The high anticonvulsant activity of quinazolines is also a consequence of their high membrane permeability[18].

**Fig. 1:**
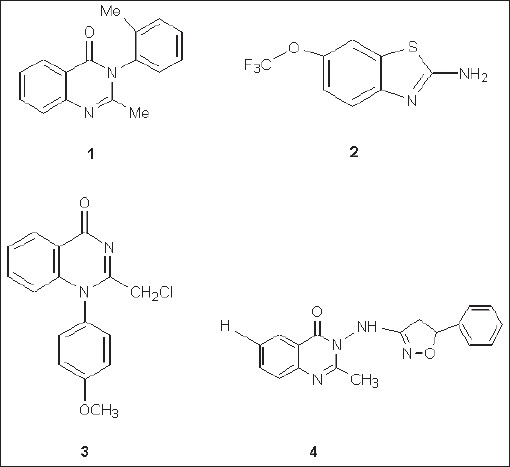
Some of potent anticonvulsant compounds. 1 is methaqualone, 2 is rilozole, 3 is 2-(chloromethyl)-1-(4-methoxyphenyl)-quinazolin-4(1*H*)-one (3) and 4 is 2-methyl-3-(5-phenyl-4,5-dihydroisoxazoli-3-ylamino)quinazolin-4(3*H*)-one

Earlier, we had reported[19,20] synthesis and antimicrobial activity of Schiff bases of 3-amino-6,8-dibromo-2-phenyl-quinazolin-4-(3*H*)-ones (9a-l). Taking cognizance of the reported high anticonvulsant activity of the quinazolines, it was decided to synthesize the Schiff bases of 3-amino-quinazolines according to the earlier reported procedure[19,20] and evaluate their anticonvulsant activity.

Synthesis of Schiff bases (9a-l) of 3-amino-6,8-dibromo-2-phenyl-quinazolin-4(3*H*)-ones[19,20] (8) with various substituted aldehydes was achieved by refluxing their equimolar dry ethanol solution for 6 h. The Schiff bases (9a-l) were filtered, dried and recrystallised from absolute ethanol (fig. 2). All compounds (9a-l) were analyzed using detailed spectroscopic (IR, ^1^H NMR, ^13^C NMR, Mass) and elemental analysis.

**Fig. 2:**
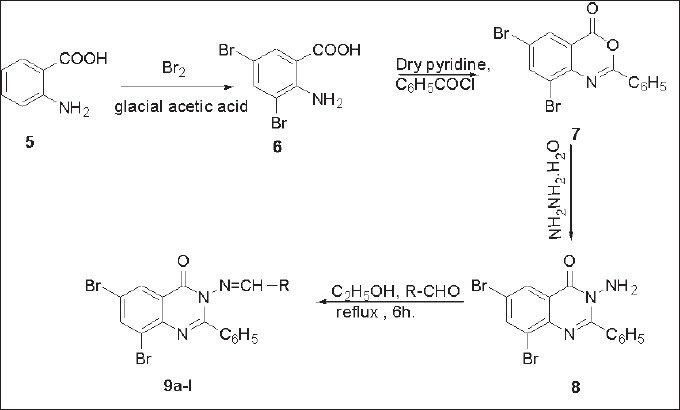
Synthesis of Schiff bases of 3-amino-6,8-dibromo-2-phenyl quinazolin-4(3*H*)-ones 9(a-l) Synthesis of Schiff bases of 3-amino-6,8-dibromo-2-phenyl quinazolin-4(3*H*)-ones 9a-l from reaction of 8 with different aldehydes a-l, (a) R= Ph-, (b) R= *p*-MeO-Ph-, (c) R= *o*-OH-Ph-, (d) R= p-N(CH_3_)_2_-Ph-, (e) R= *m*-NO_2_-Ph-, (f) R= *p*-Me-Ph-, (g) R= *p*-OH-Ph-, (h) R= *p*-Cl-Ph-, (i) R= *p*-NO_2_-Ph-, (j) R= *m*,m,*p*-(OCH_3_)_3_-Ph-, (k) R= p-OH,m-OMe-Ph-, (l) R=-CH=CH-Ph

The animal study protocols were approved by Institutional Animal Ethics Committee’s (IAEC) approval. Anticonvulsant activity of all compounds (9a-l, Table 1) was evaluated by maximal electroshock (MES) method[21]. Swiss mice (n=6) of either sex selected by random sampling technique were used for the study. Phenytoin at the dose of 10 mg/kg (i.p.) was administered as standard drug for comparison. The test compounds were suspended in polyethylene glycol in the ratio of 1:9/ml in water and were given i.p. at doses of 100-200 mg/kg body weight. Dosing volume was 0.25 ml per 25 g. The animals were held at suitable position and corneal electrodes were placed on the cornea of the mice and applied 50 mA current for 0.2 sec after half an hour administration of the test compounds. Then the time spent by animals in each phase of convulsion was recorded. Animals in which extensor response was abolished were taken as protected mice. Compound 9l (82.74 %) was found to possess high anticonvulsant activity which is followed by 9g (81.61 %), 9i (81.48 %), 9k (81.48 %), 9j (80.29 %) at the dose of 100 mg/kg. Moderate anticonvulsant activity was observed for the compounds 9e (79.03%) followed by 9d (79.01 %), 9f (77.77 %), 9h (77.77 %), 9c (74.07 %) and 9b (72.88 %). Compound 9a showed very low anticonvulsant activity of (68.14%) at the dose level of 100 mg/kg.

**TABLE 1:** ANTICONVULSANT ACTIVITY OF COMPOUNDS

Compounds	Dose (mg / kg)	Flexion phase		Extensor phase	
		Mean± SEM^a^	% protection	Mean± SEM^a^	% protection
9a	100	5.66±0.4375	27.99	4.3±0.21875	68.14
	200	4.62±0.3125	35.52	3.6±0.21875	73.33
9b	100	5.66±0.4375	21.01	3.66±0.21875	72.88
	200	4.82±0.3125	32.73	3.50±0.21875	74.07
9c	100	5.66±0.4375	27.99	4.3±0.21875	74.07
	200	4.62±0.3125	35.52	3.6±0.21875	76.29
9d	100	5.66±0.4375	21.01	3.66±0.21875	79.01
	200	4.82±0.3125	32.73	3.50±0.21875	80.59
9e	100	4.5±0.21875	37.2	3.5±0.21875	79.03
	200	3.74±0.2187	48.36	3.2±0.15625	82.07
9f	100	4.5±0.21875	37.2	2.83±0.15625	77.77
	200	3.9±0.21875	45.57	2.62±0.15625	79.25
9g	100	5.33±0.3125	25.62	2.83±0.15625	81.61
	200	4.9±0.3125	31.62	2.42±0.09375	83.70
9h	100	5.0±0.3125	30.22	3.0±0.15625	77.77
	200	4.8±0.3125	33.01	2.8±0.15625	79.25
9i	100	5.3±0.3125	26.03	2.5±0.09375	81.48
	200	4.98±0.3125	30.50	2.2±0.09375	83.70
9j	100	5.83±0.4375	18.57	3.0±0.15625	80.29
	200	5.1±0.3125	28.83	2.8±0.15625	82.96
9k	100	5.66±0.4375	20.94	2.5±0.09375	81.48
	200	5.22±0.3125	27.15	2.2±0.09375	84.44
9l	100	6.5±0.4027	9.21	2.66±0.15625	82.74
	200	5.31±0.3125	25.90	2.30±0.09375	84.07
Phenytoin	10	4.8±0.3125	32.48	0.00±0.00	100
Control	-	7.16±0.5625	-	13.5±0.6875	-

a Significant error mean method: significant differences with respect to control was evaluated by ANOVA.

Structure activity relationship (fig. 3, Table 2) for all the compounds was developed on the % protection data of extensor phase at dose of 100 mg/kg. Compounds 9l, g, i, bearing CH=CH-Ph, -p-OH-Ph, -p- NO_2_-Ph moieties, showed maximum % protection of 82.74, 81.61 and 81.48. Moderate % protection of 79.01, 77.77, 74.07 and 72.88 was observed for compounds 9d, f, c, b, respectively, possessing electron donating groups such –*p*-N(CH_3_)_2_-Ph, -*p*-Me-Ph, -*o*-OH-Ph, -*p*-OMe-Ph. However, very low anticonvulsant activity was observed for 9a bearing unsubstituted aromatic ring.

**TABLE 2:** STRUCTURE ACTIVITY RELATIONSHIP

R	Activity
-CH=CH-Ph, -*p*-OH-Ph, -NO_2_-Ph	High
-N(CH_3_)_2_, -Me-Ph, -*o*-OH-Ph, -OMe-Ph	Moderate
Ph	Low

**Fig. 3:**
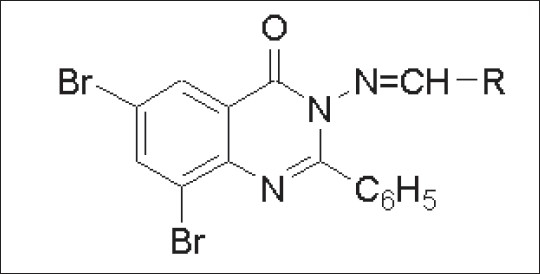
Quinazolone schiff base

In conclusion, the present study indicates that compounds 9l, g, i Possessing styryl, *p*-OH-Ph or -*p*-NO_2_-Ph were found to posses high anticonvulsant activity, which may be attributed extended conjugation in case of 9l or hydrogen bonding ability of para-substituent as in the case of 9g, i. While the compounds 9b, c, d, f bearing electron donating groups (-N(CH_3_)_2,_-Me, -*o*-OH, -OMe), display moderate activity; among the latter para-substituted compound (9d) is most active thereby confirming our earlier inference. On the other hand compound bearing unsubstituted phenyl group showed low activity. These compounds shall serve as ‘Lead’ molecules for further development.
